# Microglia and Macrophages Differentially Modulate Cell Death After Brain Injury Caused by Oxygen-Glucose Deprivation in Organotypic Brain Slices

**DOI:** 10.1002/glia.22478

**Published:** 2013-02-13

**Authors:** Sylvie Girard, David Brough, Gloria Lopez-Castejon, James Giles, Nancy J Rothwell, Stuart M Allan

**Affiliations:** Faculty of Life Sciences, University of ManchesterManchester, United Kingdom

**Keywords:** microglia, brain injury, inflammation, macrophages

## Abstract

Macrophage can adopt several phenotypes, process call polarization, which is crucial for shaping inflammatory responses to injury. It is not known if microglia, a resident brain macrophage population, polarizes in a similar way, and whether specific microglial phenotypes modulate cell death in response to brain injury. In this study, we show that both BV2-microglia and mouse bone marrow derived macrophages (BMDMs) were able to adopt different phenotypes after LPS (M1) or IL-4 (M2) treatment *in vitro*, but regulated cell death differently when added to mouse organotypic hippocampal brain slices. BMDMs induced cell death when added to control slices and exacerbated damage when combined with oxygen–glucose deprivation (OGD), independently of their phenotype. In contrast, vehicle- and M2-BV2-microglia were protective against OGD-induced death. Direct treatment of brain slices with IL-4 (without cell addition) was protective against OGD and induced an M2 phenotype in the slice. *In vivo,* intracerebral injection of LPS or IL-4 in mice induced microglial phenotypes similar to the phenotypes observed in brain slices and in cultured cells. After injury induced by middle cerebral artery occlusion, microglial cells did not adopt classical M1/M2 phenotypes, suggesting that another subtype of regulatory phenotype was induced. This study highlights functional differences between macrophages and microglia, in response to brain injury with fundamentally different outcomes, even if both populations were able to adopt M1 or M2 phenotypes. These data suggest that macrophages infiltrating the brain from the periphery after an injury may be cytotoxic, independently of their phenotype, while microglia may be protective.

## INTRODUCTION

Microglia are the resident immune cell population of the brain and are implicated in immune surveillance of normal brain parenchyma by continuously scanning for signs of danger (Marín-Teva et al.,^2012^; Prinz and Mildner,^2011^). After brain injury microglia are activated rapidly, characterized by increased proliferation and phagocytic activity, leading to their regulation of inflammatory processes (Davies et al.,^1998^; Denes et al.,^2007^; Hanisch and Kettenmann,^2007^). The contribution of peripheral macrophages to the population of microglia is minimal under normal conditions, although some extravasate into the brain parenchyma after injury, which is associated with breakdown of the blood brain barrier (BBB) (Tanaka et al.,^2003^; Vallieres and Sawchenko,^2003^). It is not clear whether infiltrating macrophages exert similar activities to resident microglia, and this is a question that is difficult to resolve since they express the same epitopes and cannot be dissociated by immunological methods (David and Kroner,^2011^; Hanisch and Kettenmann,^2007^).

One of the most important characteristics of macrophages with respect to how they shape inflammatory responses is their capacity to adopt a spectrum of different phenotypes depending on the stimulus, its duration, and the environment (Biswas and Mantovani,^2010^; Gordon and Taylor,^2005^). This process, called polarization, has been increasingly studied in recent years and has led to an improved understanding of the contribution of macrophages to several pathologies. Classical activation (M1) is typically induced by exposure to bacterial products (e.g. lipopolysaccharide, LPS) or proinflammatory cytokines (e.g. interferon-γ). Alternative activation (M2) is induced by anti-inflammatory cytokines such as interleukin (IL)-4 and IL-13. Several subsets of M2 macrophages have been described with overlapping markers and functions (Gordon and Taylor,^2005^; Biswas and Mantovani,^2010^). There is evidence that microglia may also polarize to different phenotypes, which may account for their dual role after injury. For instance, anti-inflammatory treatment after acute brain injury decreases microglial activation and is neuroprotective (Franco et al.,^2012^; Yrjanheikki et al.,^1998^), but ablation of microglial proliferation is associated with increased damage (Lalancette-Hebert et al.,^2007^). A better understanding of microglial phenotype after brain injury is required and will allow us to determine whether it is possible to modulate their phenotype and subsequently the extent of cell death.

The objective of this study was to investigate the capacity of microglia to adopt M1 or M2 phenotypes compared with peripheral macrophages, and to determine how their polarized status modulates cell death after acute brain injury. We used organotypic brain slices *in vitro* with exogenous addition of macrophages or BV2-microglia, and found that both cell types differentially modulated cell death after acute brain injury. We further showed that endogenous microglia, both *in vitro* and *in vivo*, could also polarize to different phenotypes, and that M2, and nonpolarized microglia, were protective after injury. These data highlight important differences between macrophages and microglia in response to brain injury and suggest that microglial phenotype could represent a therapeutic target for the treatment of brain injuries such as stroke.

## MATERIALS AND METHODS

All animal experiments were carried out under the UK Animals (Scientific Procedures) Act, 1986. All chemicals used were from Sigma Aldrich (Dorset, UK) unless otherwise specified.

### Bone Marrow Derived Macrophages, BV2-Microglia, and Primary Mixed Glial Cells

Bone marrow cells were isolated from the femur and tibia of 8- to 10-week old male C57BL/6 mice (Harlan, Hillcrest, UK) which were sacrificed by CO_2_ overdose, as previously described (Lopez-Castejón et al.,^2010^). Cells were resuspended and cultured in 70% Dulbecco's Modified Eagle Medium (DMEM) supplemented with 10% fetal bovine serum (FBS), 100 U/mL penicillin, 100 μg/mL streptomycin, and 30% of L929-cell conditioned media. Resulting BMDMs were used after 6–8 days. This protocol was previously shown to result in >95% purity of macrophages (Lopez-Castejón et al.,^2010^). In addition, we performed immunofluorescence (IF) (see protocol below) and found that >98% of the cells were positive for CD68 and Iba1 (*N* = 3, data not shown). The BV2 microglial cell line was maintained in RPMI (Life Sciences, Paisley, UK) supplemented with 10% FBS, 100 U/mL penicillin and 100 μg/mL streptomycin. Cells were used when 80–90% confluent. Cells were maintained at 37°C, 5% CO_2_ for all experiments. For polarization, cells were seeded in six wells plates (VWR, Lutterworth, UK) at a density of 1 × 10^6^ cells/mL and treated the following day. Murine mixed glial cells were prepared from 2- to 3-day old C57BL/6 mice as previously described (Pinteaux et al.,^2002^). Briefly, cerebral hemispheres were dissected and meninges removed. Cells were dissociated and cultures using DMEM supplemented with 10% FBS, 100 U/mL penicillin, and 100 μg/mL streptomycin. Media was changed after the first 5 days and every other day after. Cells were maintained at 37°C, 5% CO_2_ for all experiments. Cells were seeded into 24 wells plates (VWR, Lutterworth, UK) and treated when they reached approximately 90% confluency (10–12 days).

### Organotypic Hippocampal Slice Cultures

Organotypic hippocampal slice cultures (OHSC) were prepared based on the protocol described previously (Stoppini et al.,^1991^) with slight modifications. Brains were taken from 6- to 7-day-old C57BL/6 mice (killed as above), embedded in 1% low-melting agarose (Fisher Scientific, Loughbourough, UK) and transverse sections, 300 μm thick, were cut using a vibrating microtome (Leica Microsystems, Milton Keynes, UK). Hippocampi were dissected out and transferred to 0.4 μm porous membrane inserts (Millipore, Watford, UK). Four hippocampal sections were plated on each 30 mm insert in a 6-well plate containing 1 mL of media (50% HEPES buffered-MEM, 25% heat inactivated horse serum, 25% HBSS with 2 mM glutamine, 100 U/mL penicillin, and 100 μg/mL streptomycin, pH 7.2). OHSC were maintained in an incubator at 37°C, 5% CO_2_. A complete media change was made the next day and every other day until treatment. On Day 6, OHSC were treated in serum-free media with or without prior exposure to oxygen–glucose deprivation (OGD). OGD was induced by OHSC transfer to DMEM without glucose (Life Sciences, Paisley, UK), bubbled with N_2_ for 5 min before use. The plates were then maintained at 5% CO_2_, 1% O_2_/N_2_ at 37°C in an OGD-chamber (Coy Laboratories, MI) for 45 min. Reperfusion was achieved by transferring the OHSC to serum-free media at 5% CO_2_, 37°C. Treatments were added directly to the media at reperfusion, and OHSC were incubated for 24 h before assessment of cell death or processed for RNA extraction.

### Treatments and Exogenous Cell Addition to OHSC

BMDMs, BV2, mixed glial cells, or OHSC were treated with 1 μg/mL lipopolysaccharide, (LPS, *E. coli,* 026:B6), 20 ng/mL IL-4 (Peprotech, London, UK) or vehicle (PBS). BMDMs or BV2-microglia cells were treated for 24 h, removed (as described earlier) and resuspended in OHSC serum-free media. Cells were added on top of the OHSC within 15 min of reperfusion at a density of 2.5 × 10^4^ cells/slice. This number of cells was selected based on published studies (Neumann et al.,^2006^; Zhou et al.,^2011^).

### Cell Death Assessment

Cell death was determined by propidium iodide (PI) incorporation. PI was added to the media (10 μg/mL) and incubated for 30 min before being washed with PBS and fixed for 10 min in 4% paraformaldehyde (PFA). OHSC were cut from the insert and mounted using DAPI-containing mounting medium (Life Sciences, Paisley, UK). Pictures were taken from whole hippocampus, and PI fluorescence intensity was determined using Image J (NIH Image, US). PI intensity results are expressed as fold increase versus their paired control. *N* = 16–20 slices from at least 4 independent experiments in each condition.

### RNA Extraction and Quantitative Reverse Transcriptase PCR

Total RNA was extracted from BMDMs, BV2-microglia, mixed glia, and OHSC using Trizol (Life Sciences, Paisley, UK) following the manufacturer's instructions. RNA was reverse transcribed using M-MLV RT, oligo-dT, RNase out, and dNTPs (all from Life Sciences, Paisley, UK). Detailed methods for the qPCR was previously described (Lopez-Castejón et al.,^2011^). All the primers used were from Qiagen (Quantitect Primers Assay, Crawley, UK), and a fast real-time PCR system (7900 HT, Applied Biosystem, Warrington, UK) was used. Four markers of M1 polarization (i.e. interleukin (IL)-1β, inducible nitric oxide synthase (iNOS), IL-12β, and Nlrp3 (NACHT, LRR and PYD domains-containing protein 3)) and four markers of M2 polarization (i.e. Arginase1, mannose receptor C type 1 (Mrc1), Resistin-like molecule α (Retnla/Fizz1), chitinase-like molecule (YM1)) were analyzed. These markers were selected based on their specificity for M1 or M2 phenotype as detailed previously (Lopez-Castejón et al.,^2010^). The expression of four housekeeping genes was also analyzed (i.e. glyceraldehyde-3-phosphate dehydrogenase (Gapdh), succinate dehydrogenase complex, subunit A (Sdha), tyrosine 3-monooxygenase/tryptophan 5-monooxygenase activation protein, zeta polypeptide (Ywhaz), and hypoxanthine guanine phosphoribosyl transferase (Hprt)). No significant differences were observed between the housekeeping genes across the experimental conditions (data not shown). The relative gene expression of the phenotype markers was normalized to GAPDH, and the levels of expression in each of the conditions determined as fold increase compared with vehicle-treated control (*N* = 3–5).

### Surgical Procedures and Tissue Processing

Eight- to 10-week old male C57BL/6 mice were anesthetized using isoflurane (4% induction, 1.5% maintenance in O_2_ 0.2 L/min, N_2_O 0.4 L/min) (Abbott Laboratories, Maidenhead, UK). For intracerebral (i.c.) injection, the animals were placed in a small animal stereotaxic frame (Stoelting, IL). The skull was exposed and a small-hole craniotomy performed. Mice were injected i.c. with 1 μL of PBS (vehicle) (*N* = 4), LPS (O127:B8, 4 mg/mL) (*N* = 4), or IL-4 (0.1 mg/mL, Peprotech, London, UK) (*N* = 4), using a glass micro-needle (0.5 μL/min, Drummond Scientific Company, PA). Injection coordinates from bregma: anterior-posterior, 0.0 mm; lateral, 2.0 mm; deep, 2.5 mm. For transient middle cerebral artery occlusion (tMCAo), core body temperature was monitored and maintained at 37°C using a homeothermic blanket (Harvard Apparatus, Kent, UK). The bifurcation of the external and internal carotid artery was exposed and a silicon rubber-coated monofilament (diameter: 0.21 ± 0.02 mm; Doccol, CA) was inserted through the external carotid artery and advanced up to within the internal carotid artery to occlude the MCA. Blood flow was monitored using a laser Doppler probe (Moor Instruments, Devon, UK). Reperfusion was induced by removing the filament either 30 or 60 min after occlusion (*N* = 4/occlusion time). In sham animals the filament was removed immediately and the animals maintained under anesthesia for 60 min (*N* = 4). Twenty-four hours after i.c. injection or tMCAo animals were anesthetized using isoflurane (4% in O_2_ 0.2 L/min, N_2_O 0.4 L/min) and transcardially perfused with 0.9% saline followed by 4% PFA. Brains were removed, postfixed overnight at 4°C in 4% PFA/20% sucrose, and transferred to 20% sucrose. Coronal sections, 25 μm thick, were cut using a sledge microtome (Bright Instruments, Cambridge, UK) and transferred to cryoprotectant solution (30% ethylene glycol, 20% glycerol in PBS) and stored at −20°C until used.

### Immunofluorescence

Free-floating sections (20 μm thick) were washed in PBS and incubated for 30 min in blocking solution (PBS containing 1% donkey serum (Serotec, Oxford, UK), 2% bovine serum albumin and 0.5% Triton X-100). Sections were then incubated overnight at 4°C in blocking solution with primary antibody (Ab) (rabbit anti-ionized calcium binding adaptor molecule 1 (Iba1), Wako Chemicals, VA; rat anti-CD68, Serotec, Oxford, UK; goat anti-IL-1β, R&D System, Abingdon, UK; mouse anti-iNOS, R&D System, Abingdon, UK; rabbit anti-Ym1, StemCell Technologies, Grenoble, France; rat-anti-CD206, Serotec, Oxford, UK). Sections were washed in PBS and incubated with secondary Ab in blocking solution (anti-goat Alexa-488; anti-rat Alexa-594; anti-mouse Alexa 488; anti-rabbit Alexa-594; anti-rat Alexa-488, Life Sciences, Paisley, UK) for 2 h. Sections were washed with PBS, mounted onto Superfrost Slides (Fisher Scientific, Loughbourough, UK) and coverslipped with Prolong antifade medium containing Dapi (Life Sciences, Paisley, UK).

### Statistical Analysis

Data are presented as means ± standard error of the mean (SEM). One-way or two-way analysis of variance (ANOVA) with Bonferroni post-test was performed (see figure legends for details) using GraphPad Prism (Version 5.0, GraphPad Software, CA). Data were considered significant when *P* < 0.05.

## RESULTS

### BMDMs, BV2-Microglia, and Mixed Glial Cells Adopt M1 and M2 Phenotypes *In Vitro*

We first compared the capacities of BMDMs and BV2-microglial cells to change phenotype after treatment with typical polarising stimuli (Biswas and Mantovani,^2010^). Classically activated phenotype, M1, was induced by LPS, and alternative activation, M2, was induced by treatment with IL-4 (Table 1). Genes studied were selected for their specificity for each phenotype (M1 or M2) based on previous study (Lopez-Castejón et al.,^2010^). Treatment with LPS for 4 or 24 h induced upregulation of M1 phenotype markers (i.e. IL-1β, iNOS, IL-12β) in BMDMs and BV2-microglia. NLRP3 expression, another marker of M1 phenotype, was also significantly upregulated at 4 and 24 h in BMDMs, and at 4 h in BV2-microglia (Table 1). Treatment with IL-4 induced expression of M2 genes (i.e. Arg1, Ym1, and MRC1) in both BMDMs and BV2-microglia, with greatest levels seen after 24 h. The expression of Fizz1, another marker of M2 status was induced in BMDMs but was not detectable in BV2-microglia. Although there were differences in the levels of induction of several of the genes studied between BMDMs and BV2-microglia, there was still a net change in the phenotype, either M1 or M2, induced in both cell types (Table 1). Thus, both BV2-microglia and BMDMs were essentially able to adopt both M1 and M2 activated phenotypes. To confirm the relevancy of the BV2-microglial cell line to primary microglia, we studied the expression of the same markers of phenotype in primary mixed glial cells after treatment with M1 or M2 inducers, LPS and IL-4 respectively for 4 or 24 h. Similarly to BV2-microglia, LPS significantly induced the selective upregulation of M1-specific genes in primary mixed glial cells (i.e. IL-1β, iNOS, IL-12β, NLRP3) at both 4 and 24 h (Table 2). Treatment with IL-4 significantly induced M2-specific genes (i.e. Arg1, YM1, Fizz1), mainly at 24 h (Table 2) which was also the case in BV2-microglia (Table 1). Because of the low yield of pure primary microglia, the necessity to minimize animal use according to the UK Animals (Scientific Procedures) Act, 1986, and to the similarities between BV2-microglia and primary mixed glial cells to adopt both M1 and M2 phenotype, the rest of the experiments were performed with BV2-microglia. Treatment for 24 h with LPS (M1) and IL-4 (M2) was used to induce the different phenotypes for all subsequent experiments.

**TABLE 1:** Expression of Markers of Phenotype by BMDM and BV2-Microglia

4 h		LPS		IL-4	
		BMDM	BV2-Microglia	BMDM	BV2-Microglia
M1	IL-1β	1492 ± 590^b^,^d^	4635 ± 2124^b^	0.4 ± 0.2	1.1 ± 0.6
	iNOS	3221 ± 1295^b^,^f^	422 ± 296	0.9 ± 0.4	5.1 ± 3.8
	IL-12β	865.0 ± 437.7^a^	479 ± 197^a^	0.1 ± 0.1	1.0 ± 0.5
	NLRP3	10.9 ± 5.1^a^,^f^	57.7 ± 21.7^b^	0.3 ± 0.2	0.7 ± 0.4
M2	Arg1	1.7 ± 0.8	5.5 ± 4.3	128.0 ± 43.6^c^	62.1 ± 51.6
	Ym1	13.2 ± 12.0	1.7 ± 0.9	109.0 ± 98.3	6.7 ± 2.5^a^
	MRC1	1.0 ± 0.7	1.0 ± 0.4	4.9 ± 3.9	2.1 ± 0.7
	Fizz1	0.2 ± 0.1	ND	3072 ± 1539^a^	ND

24 h		LPS		IL-4	
		BMDM	BV2-Microglia	BMDM	BV2-Microglia
M1	IL-1β	1622 ± 1006^a^,^d^	86.5 ± 58.5^a^	0.2 ± 0.1	4.1 ± 2.5
	iNOS	5365 ± 2810^b^,^e^	113.7 ± 43.0^c^	9.1 ± 5.2	13.6 ± 6.9
	IL-12β	437 ± 242^a^	1310 ± 660^a^	0.4 ± 0.1	12.2 ± 9.9
	NLRP3	18.3 ± 10.2^a^,^e^	0.8 ± 0.7	0.6 ± 0.2	2.0 ± 0.7
M2	Arg1	1868 ± 974^b^,^e^	0.5 ± 0.4	325 ± 202	118.0 ± 53.8^a^
	Ym1	6.2 ± 5.4	0.2 ± 0.2	374 ± 264^a^	205 ± 110^a^
	MRC1	0.1 ± 0.1	0.1 ± 0.1	3.9 ± 2.0^a^	16.2 ± 7.5^a^,^d^
	Fizz1	0.3 ± 0.1	ND	23518 ± 8287^a^	ND

Fold increase as compared to vehicle-treated control, mean ± SEM, *N* = 5.

a *P* < 0.05;

b *P* < 0.01;

c *P* < 0.001 One-way ANOVA, Bonferroni post test for comparison with control;

d *P* < 0.05,

e *P* < 0.01,

f *P* < 0.001 Two-way ANOVA, Bonferroni post-test to compare BMDM and BV2-microglia; ND, non-detectable.

**TABLE 2:** Expression of Markers of Phenotype in Mixed Glial Cells

		4 h		24 h	
			
		LPS	IL-4	LPS	IL-4
M1	IL-1β	843.6 ± 222.5^c^	0.4 ± 0.1^c^	250.6 ± 75.3^b^	0.9 ± 0.3
	iNOS	2912 ± 963.3^c^	1.2 ± 0.4	4951 ± 1301^b^	3.0 ± 1.2
	IL-12β	31042 ± 9357^b^	1.8 ± 0.8	764.5 ± 215.6^b^	2.8 ± 2.1
	NLRP3	65.3 ± 13.5^c^	2.0 ± 0.2	3.7 ± 0.5^a^	2.4 ± 0.8
M2	Arg1	2.1 ± 0.9	17.6 ± 4.6^c^	36.1 ± 16.6	527.1 ± 221.3^a^
	Ym1	0.4 ± 0.1	2.7 ± 1.2	0.6 ± 0.3	123.9 ± 52.8^a^
	MRC1	0.6 ± 0.2	1.8 ± 0.4	0.01 ± 0.004	3.0 ± 1.1
	Fizz1	3.1 ± 1.9	27.8 ± 8.6^a^	3.3 ± 2.4	8983 ± 2536^c^

Fold increase as compared to vehicle-treated control, Mean ± SEM, *N* = 3.

a *P* < 0.05;

b *P* < 0.01;

c *P* < 0.001 one-way ANOVA, Bonferroni post-test for comparison with vehicle-treated control.

### Exogenous Macrophages and Microglia Differentially Modulate Cell Death After Brain Injury *In Vitro*

To assess the role of macrophages and BV2-microglia and their phenotypes on neuronal cell death induced by ischaemia, we used OGD-induced damage in OHSC with or without exogenous addition of polarized BMDMs or BV2-microglia.

**FIGURE 1:**
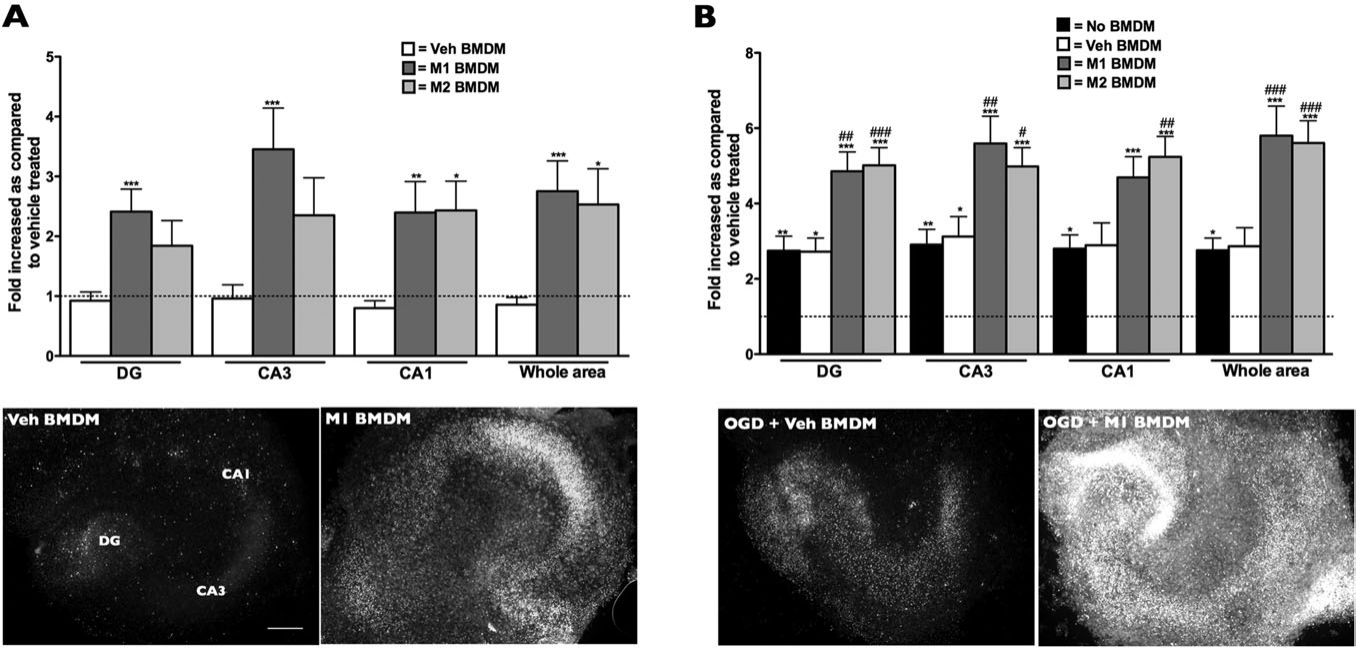
The effects of BMDM addition to OHSC. Addition of M1 or M2 -BMDMs to control OHSC induced cell death as seen by the increased PI uptake throughout the hippocampus (**A**). Exacerbation of cell death was seen after combined exposure to OGD with either M1 or M2 BMDMs added at reperfusion (**B**). Data are presented as mean ± SEM, *N* = 16–20 slices from at least four independent sets of experiments, *P < 0.05; **P < 0.01; ***P < 0.001 compared with control and ^#^*P* < 0.05; ^##^*P* < 0.01; ^###^*P* < 0.001 compared with OGD. One-way ANOVA, Bonferroni post-test. Scale bar: 500 μm.

Addition of M1 BMDMs to control slices in the absence of OGD induced cell death (2.75 ± 0.50 fold increase as compared with control slices), as seen by increased PI uptake throughout the hippocampus (Fig. 1A). Addition of M2 BMDMs also induced cell death detected as a significant increase in PI uptake in the CA1 region (Fig. 1A). This increased cell death mediated by exogenous M1 or M2 BMDMs in control slices was similar to the cell death induced by exposure to OGD alone (2.76 ± 0.32 for OGD-alone, 2.75 ± 0.51 for M1, and 2.53 ± 0.60 for M2-BMDM addition onto control slices, fold increase over vehicle treated slices without cell addition, Fig. 1A,B). When OGD was combined with the exogenous addition of M1 or M2 BMDMs, added at the time of reperfusion, PI uptake was significantly exacerbated in the DG and CA3 by M1-BMDMs, and throughout the hippocampus by M2-BMDMs (Fig. 1B). Addition of vehicle-treated BMDMs did not modulate PI uptake further than OGD itself.

**FIGURE 2:**
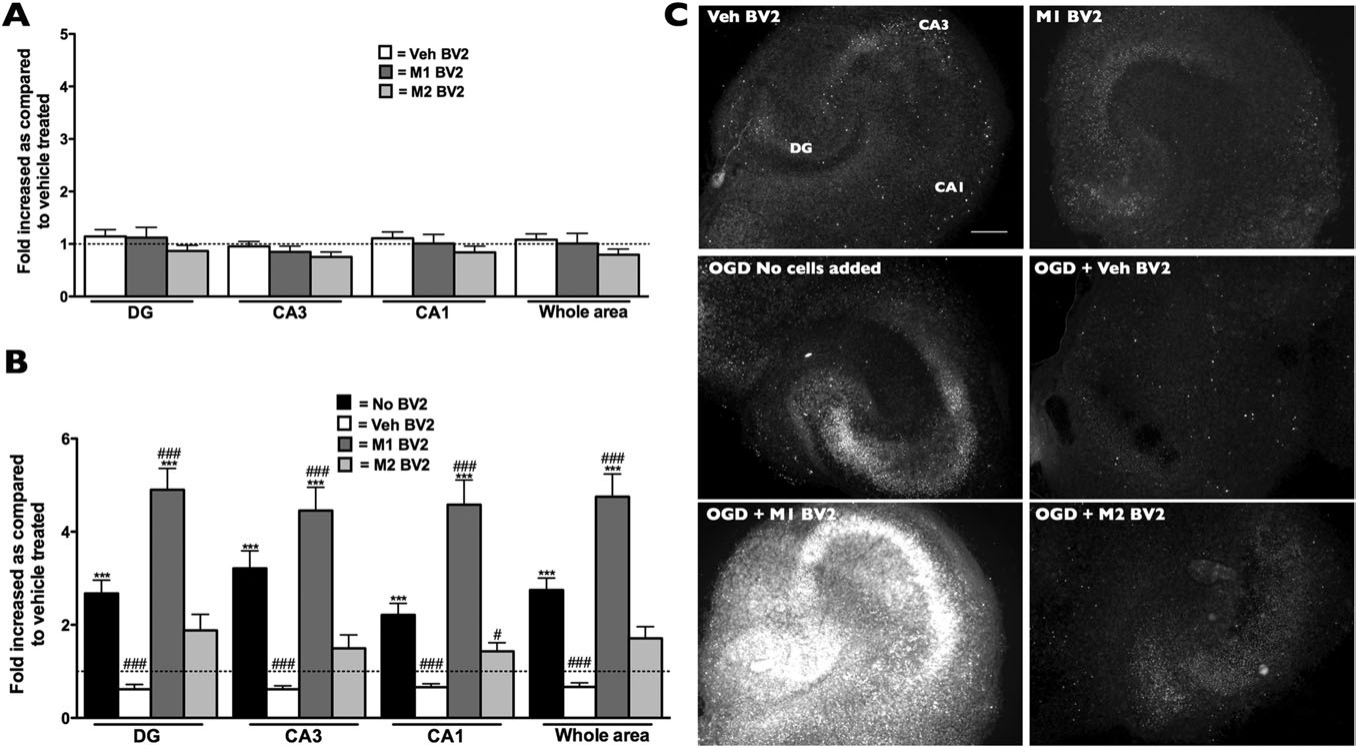
The effects of BV2-microglia addition to OHSC. Addition of M1 or M2- BV2-microglia to control OHSC did not induce any changes in cell death (**A**). After OGD-induced brain injury, addition of vehicle treated or M2- BV2-microglia was protective, as seen by the reduced PI uptake. M1-BV2-microglia exacerbated cell death (**B**). Representative example of PI uptake by OHSC (**C**). Data are presented as mean ± SEM, *N* = 16–20 slices from at least four independent set of experiments, ***P < 0.001 compared with control and ^#^*P* < 0.05; ^###^*P* < 0.001 compared with OGD. One-way ANOVA, Bonferroni post-test. Scale bar: 500 μm.

In contrast to BMDMs, addition of exogenous BV2-microglia, of any phenotype, to control brain slices in the absence of OGD did not induce cell death (Fig. 2A). After OGD, addition of vehicle treated BV2-microglia was neuroprotective throughout the hippocampus, completely abrogating cell death induced by OGD (Fig. 2B,C). Although addition of M2-BV2-microglia after OGD was not as protective as vehicle treated BV2 cells, it significantly reduced PI uptake in the CA3 region as compared with OGD alone-treated slices (Fig. 2B). Addition of M1-BV2-microglia exacerbated cell death to a similar extent to the addition of M1 BMDMs (Figs. 1B and 2B,C).

Considering that both M1 and M2 BMDMs induced cell death in brain slices, we looked at the protein levels of M1-markers which are known to be cytotoxic (i.e. NO release and IL-1β levels) to make sure that those were not induced in the M2 population and responsible for the lack of differences between both BMDM phenotypes as compared with the BV2-microglia. Nitrite release was observed only with LPS treatment, in both BMDM (71.0 ± 8.2 μM in LPS vs. 5.8 ± 1.7 μM in vehicle-treated, *P* < 0.001) and BV2-microglia (34.2 ± 1.8 μM in LPS vs. 0.6 ± 0.3 μM in vehicle-treated, *P* < 0.001), although to a lesser extent in the latter. IL-1β levels were also induced selectively by LPS in BMDM (5259 ± 1855 pg/mL in LPS vs. 66.3 ± 15.4 pg/mL in vehicle-treated, *P* < 0.05) and BV2-microglia (351.7 ± 109.4 pg/mL in LPS vs. 81.6 ± 8.8 pg/mL in vehicle-treated, *P* < 0.05).

### Endogenous Microglia in Brain Slices Adopt M1/M2 Phenotype and Modulate Cell Death Induced by OGD

**FIGURE 3:**
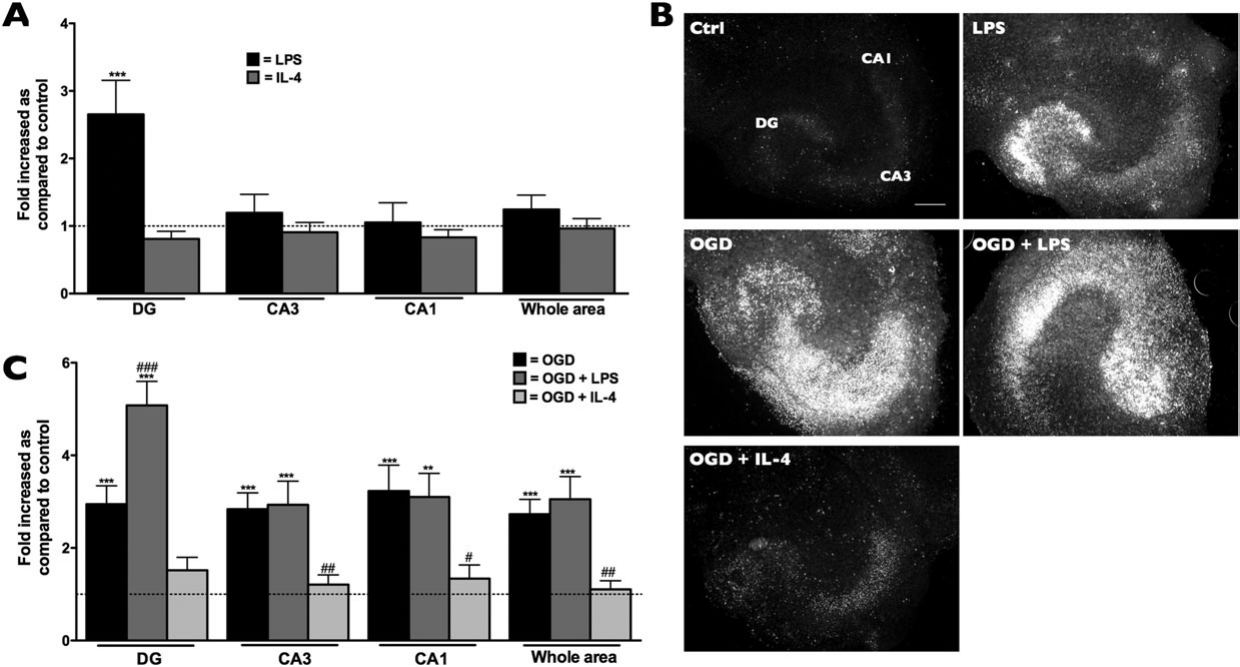
Modulation of cell death in OHSC by treatment with LPS or IL-4. Treatment of OHSC with LPS (M1-inducer) or IL-4 (M2) did not alter PI uptake in OHSC, except for the increase observed in the DG with LPS treatment (**A**). After OGD-induced injury, treatment with IL-4 was protective (**B**). Representative example of PI uptake by OHSC (**C**). Data are presented as mean ± SEM, *N* = 16–20 slices from at least 4 independent set of experiments, ***P < 0.001 compared with control and ^#^*P* < 0.05; ^###^*P* < 0.001 compared with OGD. One-way ANOVA, Bonferroni post-test. Scale bar: 500 μm.

Having determined that exogenous microglia and BMDMs have very different effects on OHSC, we next investigated whether the endogenous microglial cell population within the OHSC could adopt different phenotypes and whether this modulated cell death. OHSC were exposed to the same polarizing stimuli as used on exogenous cells at reperfusion. Treatment with LPS induced cell death in the DG of control OHSC (Fig. 3A,B). LPS also exacerbated PI uptake, only in the DG, when combined with OGD (Fig. 3B,C). In contrast, treatment with IL-4 was protective against OGD-induced cell death in the CA3 and CA1 region of the hippocampus (Fig. 3B,C).

Modulation of cell death correlated well with the expression of relevant markers of phenotype within the OHSC. Treatment with LPS induced a clear M1 gene expression profile in the slices as seen by the significantly increased expression of IL-1β, iNOS, and IL-12β. IL-4 induced a typical M2 profile of gene expression, characterized by the significant induction of Arg1, MRC1, and YM1 (Table 3). These changes in microglial phenotype were similar to that observed in exogenous BV2-microglial cells (Table 1), and mixed glial cells (Table 2) and showed the capacity of endogenous microglia to adopt different phenotypes within the brain environment. Surprisingly, OGD did not induce a clear M1 or M2 phenotype (Table 3). However, when OGD was combined with LPS, expression of IL-1β and iNOS was similar to LPS alone, but IL-1β induction was higher (Table 3). OGD plus LPS also induced the expression of M2 markers (i.e. Arg1 and YM1) and a decrease in the expression of MRC1 compared with control (Table 3). The combined exposure to OGD with a M1 stimulus (i.e. LPS) therefore induced a mixed microglial phenotype, compared with the clear M1 status induced by LPS alone. In contrast to this, the expression of all three M2 markers studied (i.e. Arg1, YM1, and MRC1) were significantly decreased in OGD plus IL-4 treated slices compared with IL-4 alone (Table 3). This indicates that although OGD itself did not induce the expression of the studied genes, it can modulate microglial phenotype.

**TABLE 3:** Expression of Markers of Phenotype in OHSC

		Vehicle	LPS		IL-4	
				
		With OGD	No OGD	With OGD	No OGD	With OGD
M1	IL-1β	1.5 ± 0.7	249.0 ± 64.8^c^	286.0 ± 36.9^c^	1.8 ± 0.3	2.0 ± 0.5
	iNOS	643 ± 360	1390 ± 370^c^	2856 ± 905^c^	2.8 ± 0.7	2.2 ± 1.0
	IL-12β	3.6 ± 3.0	27.1 ± 3.9^c^	149 ± 30^c^,^e^	3.3 ± 1.7	2.0 ± 0.7
M2	Arg1	17.3 ± 13.2	24.5 ± 5.1	55.4 ± 21.7^a^	295.0 ± 70.6^c^	69.3 ± 15.0^b^,^e^
	Ym1	0.7 ± 0.2	0.9 ± 0.1	12.1 ± 7.5^a^	22.1 ± 6.9^c^	2.9 ± 1.1^d^
	MRC1	1.4 ± 0.3	0.2 ± 0.1	0.2 ± 0.03^a^	7.8 ± 2.5^c^	2.1 ± 0.3^c^,^d^

Fold increase as compared to vehicle-treated control, mean ± SEM, *N* = 5.

a *P* < 0.05;

b *P* < 0.01;

c *P* < 0.001 One-way ANOVA, Bonferroni post test for comparison with control;

d *P* < 0.05;

e *P* < 0.01 *t*-test with Welch correction for comparison with/without OGD.

### M1 and M2 Microglial Phenotypes Can Be Induced In Vitro, but a Clear Phenotype Is Not Observed After Experimental Brain Injury

**FIGURE 4:**
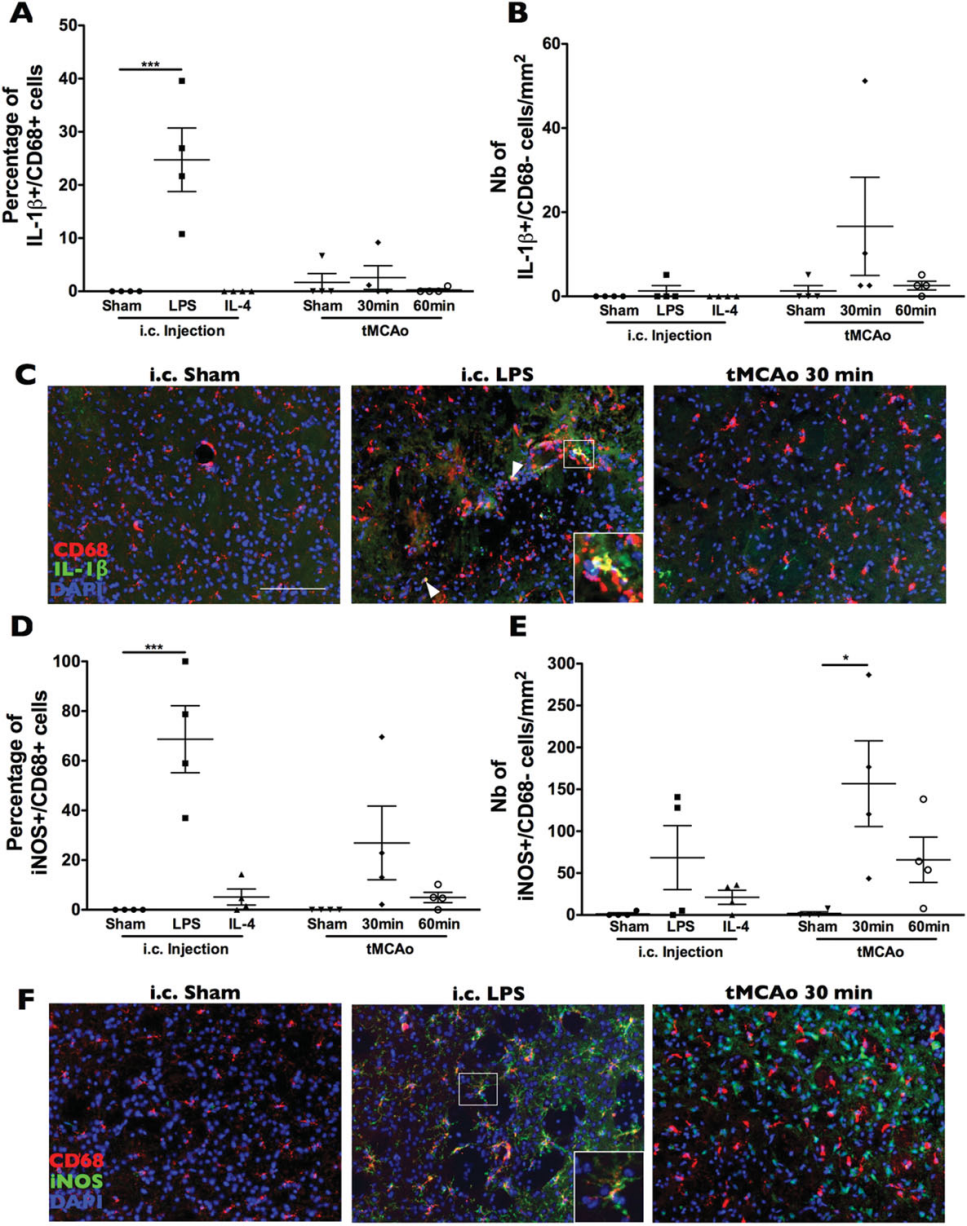
*In vivo* expression of M1 markers. i.c. injection of LPS induced expression of IL-1β in CD68+ cells (**A**) with minimal induction in CD68- cells observed only after tMCAo (**B**). Representative example of IL-1β staining localised to the injection site (within the striatum) (**C**). i.c. injection of LPS induced expression of iNOS in CD68+ cells (A) with some induction in CD68^–^ cells observed after 30 min tMCAo (B). Representative example of iNOS staining localised to the injection site or to the infarct border (within the striatum) (C). Data are presented as mean ± SEM, *N* = 4, *P < 0.05; ***P < 0.001 compared with their respective sham animals. One-way ANOVA, Bonferroni post-test. Scale bar: 100 μm. [Color figure can be viewed in the online issue, which is available at http://wileyonlinelibrary.com.]

**FIGURE 5:**
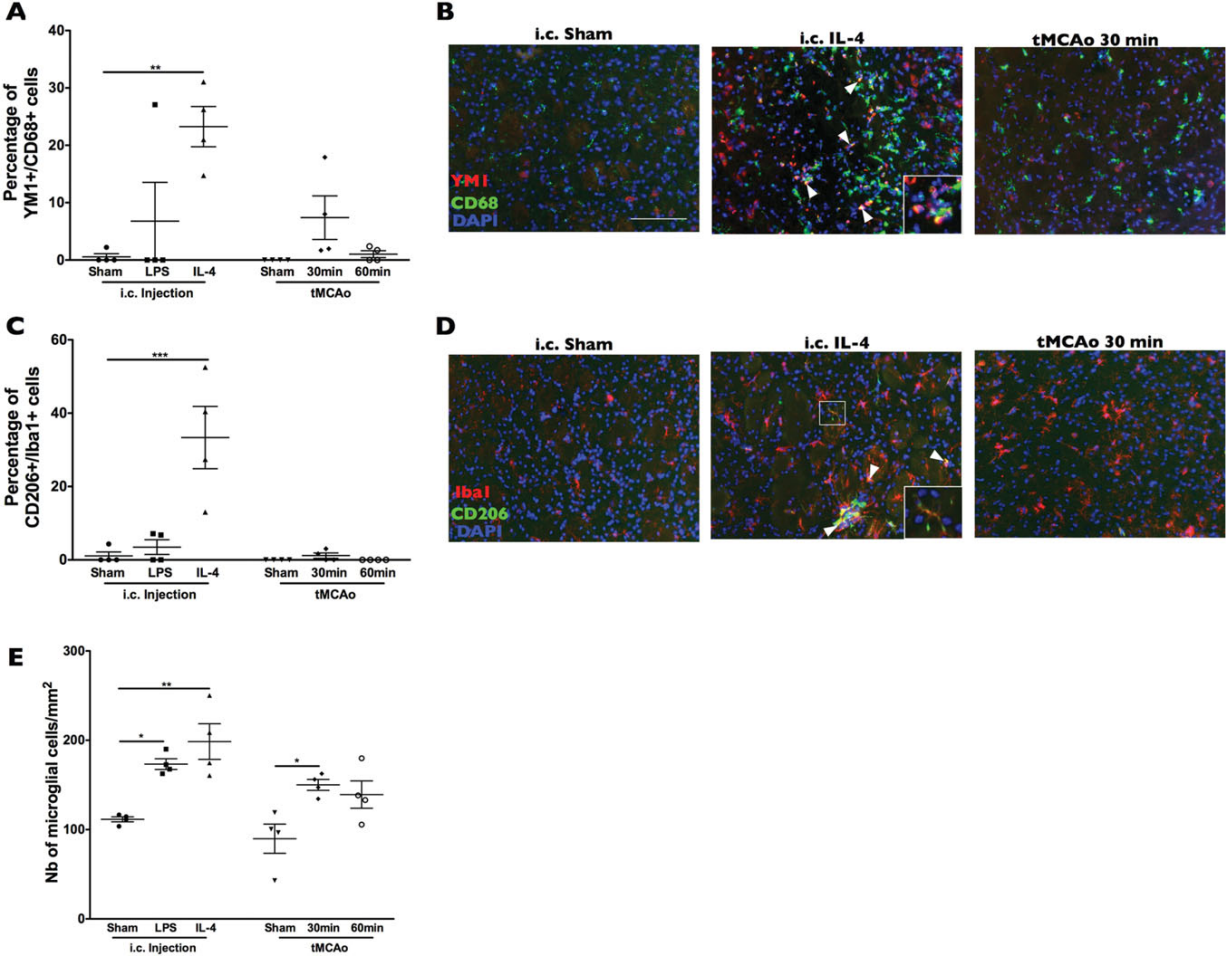
*In vivo* expression of M2 markers. i.c. injection of IL-4 induced expression of YM1 (**A**, **B**) and CD206 (**C**, **D**) in CD68+ cells. The number of microglial cells was increased by i.c. injection of IL-4 or by 30 min tMCAo (**E**). Data are presented as mean ± SEM, *N* = 4/conditions, *P < 0.05; **P < 0.01; ***P < 0.001 compared with their respective sham animals. One-way ANOVA, Bonferroni post-test. Scale bar: 100 μm. [Color figure can be viewed in the online issue, which is available at http://wileyonlinelibrary.com.]

Given that endogenous microglia within OHSC can adopt different phenotypes, we investigated if the expression of M1 and M2 markers is also induced *in vivo*, whether this was specific to microglial cells (i.e. using CD68 or Iba1 markers) and how acute brain injury modulated the expression of those markers. We first determined if CD68+ microglial cells expressed markers of M1 or M2 phenotype in response to intracerebral administration of either LPS or IL-4, and then compared the pattern of expression to a model of acute brain injury (tMCAo). Injection (i.c.) of LPS led to increased expression of IL-1β in CD68^+^ cells (Fig. 4A,C). Very few CD68^−^ cells were positive for IL-1β (Fig. 4B). LPS also induced the expression of iNOS in CD68^+^ cells although this induction was also observed in some CD68^−^ cells (Fig. 4D–F). The expression of two markers of M2 phenotype analysed (YM1 and CD206) was induced only in CD68^+^ in response to injection of IL-4 (Fig. 5). The expression of all markers, except for iNOS, was localized to the injection site (data not shown).

After tMCAo, none of the markers were significantly expressed by CD68^+^ cells (Fig. 4 and 5). Increased iNOS expression in CD68^−^ cells was observed after 30 min tMCAo (Fig. 4E,F). Even when a longer occlusion time was used (i.e. 60 min), which tripled the infarct volume (60 min: 48.4 ± 5.4 mm^3^ vs. 30 min: 17.0 ± 1.6 mm^3^, *P* < 0.001, mean ± SEM), there was no induction of any of the markers studied (Figs. 4 and 5A–D). The expression of M1 or M2 markers by microglia *in vivo* was not related to cell death, since there was no neuronal damage induced in the brain after i.c. injection of LPS or IL-4 compared with tMCAo (observed using Nissl staining, data not shown). Microglial proliferation was induced by injection (i.c.) of LPS or IL-4 and also by 30 min tMCAo (Fig. 5E). Although cell proliferation can be associated with phenotype, the fact that MCAo also induced an increased number of microglia suggests that in this case the two processes are not directly related. Overall, these *in vivo* observations correlated with the *in vitro* findings using OHSC and further confirmed that microglia are also able to adopt multiple phenotypes *in vivo*, although acute brain injury does not induce a typical M1 or M2 phenotype, at least within the time window studied.

## DISCUSSION

The capacity of macrophages to adopt different phenotypes, a process called polarization, is central to their function in diverse pathologies (Biswas and Mantovani,^2010^; David and Kroner,^2011^; Gordon and Taylor,^2005^; Sica and Mantovani,^2012^). Whether microglia can adopt different phenotypes, similarly to peripheral macrophages and whether the phenotype of both affected cell death after acute brain injury was unknown. We found that microglia exerted activities distinct from peripheral macrophages, even when both cell types adopted a similar M1 or M2 phenotype. Furthermore we showed that microglia could adopt M1 and M2 phenotypes both *in vitro* and *in vivo*, and that an M2 phenotype was protective after acute injury in brain slices.

The neuroprotective effect of BV2-microglia we report is consistent with a previous study in which the protection is specific to BV2-microglia as compared with granulocytes (Neumann et al.,^2006^). Another study also reports the protective role of primary microglia within OHSC in a model of excitotoxicity, although they did not determine the phenotype of microglia (Vinet et al.,^2012^). Furthermore, *in vivo* studies also showed the importance of microglia in protection after brain injury (Faustino et al.,^2011^; Lalancette-Hébert et al.,^2007^). The exact mechanisms are still to be defined but points towards limitation of inflammation as well as reducing oxidative stress (Faustino et al.,^2011^). Here we extend this finding by demonstrating that microglia exerted opposite effects to peripheral macrophages, which induced cell death independently of their phenotype. We also showed that exogenous addition of nonpolarized BV2-microglia was protective, and that expansion of the microglial population could therefore be a possible therapeutic mechanism. The fact that we used a microglial cell line to compare with primary BMDMs is an issue, but since primary microglia (both *in vitro*—in mixed glia and within brain slices—and *in vivo*) adopt M1 and M2 phenotypes similarly to BV2-microglia, showed that the use of this cell line is representative of primary microglia. The specificity of the neuroprotection mediated by microglia that we showed is different from that reported by a recent study, which highlighted the importance of macrophages to prevent hemorrhagic transformation (Gliem et al.,^2012^). However, in the latter study, the main population of cells recruited to the brain after injury was undifferentiated monocytes/macrophages, which then differentiated within the parenchyma. In this study, macrophages were differentiated prior to their addition onto brain slices. It is possible that macrophages are less plastic then monocytes and this might account for the differences observed. Furthermore, macrophages are reported to respond heterogeneously within a population (van Rossum et al.,^2008^). Further studies would be necessary to address the polarization capacities of macrophages at different stages of differentiation, and how this would affect their role during brain injury.

The fact that M1-BMDMs induced cell death in control brain slices, whereas M1-BV2-microglia did not, could be explained by the higher levels of IL-1β being expressed and nitrite being released in BMDMs. The differences we observed after addition onto OGD-exposed brain slices may result from a reversal of BMDMs to a cytotoxic phenotype. It is known that in spinal cord injury, M2-macrophages can revert to M1 once in contact with the injured spinal cord (Kigerl et al.,^2009^). Furthermore, conditioned media from OGD-exposed neurons can induce an M1 phenotype in microglial cells (Hu et al.,^2012^). In our experimental design, it is possible that the exogenously added M2-BMDMs onto OGD-exposed brain slices were converted towards a M1 cytotoxic phenotype, although the time frame in this study (i.e. 24 h) is short, compared with the delay (i.e. 3 days) for this effect to be observed *in vivo*. Also, vehicle treated BMDMs did not induce or exacerbate damage, showing that their phenotype was not altered by contact with the slices and this phenomenon was specific to BMDMs, as compared with M2-microglia. Furthermore, transplantation studies using chimeric mice in an Alzheimer's model, showed that “bone-marrow derived microglia” were more potent at eliminating Aβ plaques than resident microglia (Simard et al.,^2006^). Although there was no indication that bone marrow derived cells adopt a specific phenotype when in contact with the brain parenchyma, these data suggest that bone marrow derived cells are more toxic than resident microglia, which is in line with our results. However, further studies are necessary to address the specificity of microglial-mediated neuroprotection, as well as the capacity of microglia to change phenotype as compared with BMDMs, which are known to switch from one phenotype to the other (Lopez-Castejón et al.,^2010^).

The role of IL-4 in neuroprotection has been demonstrated in mouse models of Alzheimer's disease (Kiyota et al.,^2010^) and stroke (Xiong et al.,^2011^). In the latter study, IL-4 KO mice had larger infarcts, which were reversed by IL-4 administration (Xiong et al.,^2011^). However, it is still unknown whether this protection is dependent on microglial phenotype or to other actions. It was also shown that IL-4 delayed pathology progression in an experimental model of multiple sclerosis (Butovsky et al.,2006a) and promoted oligodendrogenesis (Butovsky et al.,2006b). Studies on M2 activation in macrophages after parasitic infections show that IL-4 plays a central role in the expansion of this specific population of macrophages, through local proliferation, as opposed to M1 inflammation, which is characterised by influx of cells from the circulation (Jenkins et al.,^2011^). A similar mechanism could be induced in the brain, especially since microglia are known to proliferate after brain injury (Lalancette-Hébert et al.,^2007^) and self-renewal is known to be the main mechanism of maintenance of the microglial population in the normal brain (Ajami et al.,^2007^). Our results further show that both the increased number of microglia as well as M2 polarization of endogenous microglia can be protective after *in vitro* brain injury, and that *in vivo* administration of IL-4 led to an increased number of microglia and their expression of M2 markers. Even if IL-4 did induce a M2-phenotype within brain slices after OGD, the induction was not as significant as that observed without OGD. This might reflect the high plasticity of microglia in response to multiple stimuli. A similar effect is also observed in an *in vivo* model of spinal cord injury (Kigerl et al.,^2009^). This also highlights the necessity of repeated administration of therapeutic agent (e.g. IL-4) to maintain the microglial phenotype. The fact that OGD and tMCAo did not induce a distinct phenotype in microglia, under these experimental conditions, questions the current assumption that microglial activation after injury, as observed by analysis of microglial morphology, is proinflammatory early after damage (Hanisch and Kettenmann et al.,^2007^; Marín-Teva et al.,^2012^). A recent study used a cuprizone model of demyelination to show that microglia could adopt different phenotypes, although these phenotypes were not the typical M1 or M2 (Olah et al.,^2012^), as both M1/M2 genes were induced. Further studies similar to this, using gene array may elucidate the specific microglial phenotype induced at different stages after brain injury.

Further studies are necessary to fully understand the phenotype of microglia within resting as well as injured brain. Our data suggest that OGD modulates the capacity of microglia to adopt different phenotypes, when combined with other stimuli, and alone did not induce a typical M1 phenotype. It is possible that some of the cells positive for microglial markers (CD68 or Iba1) were infiltrating macrophages in response to central injection of a particular stimulus. However, unlike tMCAo, which is associated with widespread cell death and well-characterized BBB disruption (Denes et al.,^2007^), i.c. injection of either LPS or IL-4 induced minimal cell death (assessed by cresyl violet staining, data not shown) and minimal BBB disruption (assessed by IgG immunostaining, data not shown). Furthermore, the *in vivo* results closely mirror the results obtained with the OHSC, which are completely devoid of macrophages.

In summary, our results showed functional differences between microglia and macrophages when exogenously added to OGD-exposed OHSCs. Independently of their phenotype, macrophages were detrimental in all cases, while only M1 microglia were toxic, with the other phenotypes observed offering protection. This suggests that exposure of the brain parenchyma to invading macrophages is to be avoided, and strategies to prevent this may be effective in treating neurodegenerative disease. Endogenous microglia have the capacity to adopt M1 or M2 phenotypes, to a similar extent as their exogenous counterparts used in this study, and adoption of an M2-phenotype was protective against OGD-induced cell death. However, after injury microglia adopted a new phenotype distinct from classical M1 and M2. Further work is required to determine what, if any, the functional consequences of this phenotype are. However, it is possible to induce this microglia toward a M2 phenotype, which is protective, and this may represent a new strategy for treating acute brain injury.
